# Optical Detection of Water Adulteration in Ethanol Using PCPDTBT-Conjugated Polymer Nanoparticles

**DOI:** 10.1021/acsomega.5c07266

**Published:** 2026-01-10

**Authors:** Claudio Y. Morassuti, Leandro O. Araujo, Samuel L. Oliveira, Anderson R. L. Caires

**Affiliations:** 1 Grupo de Óptica e Fotônica, 54534 Instituto de Física, Universidade Federal de Mato Grosso do Sul, Campo Grande, MS 79.070-900, Brazil; 2 Grupo de Biofabricação, 193232 Centro de Tecnologia da Informação Renato Archer, Campinas, SP 13.069-901, Brazil

## Abstract

The detection of water adulteration in ethanol biofuels is a persistent challenge due to ethanol’s high affinity for water and the limitations of conventional analytical methods. In this study, we present an innovative optical sensing strategy using conjugated polymer nanoparticles (CPNs) composed of poly­[2,6-(4,4-bis­(2-ethylhexyl)-4*H*-cyclopenta-[2,1-b;3,4-b0]­dithiophene)-*alt*-4,7-(2,1,3-benzothiadiazole)] (PCPDTBT) for rapid and sensitive quantification of water content in ethanol blends. These nanoparticles were synthesized via nanoprecipitation and exhibited dual-emission bands centered at 679 and 846 nm, corresponding to nanoparticle fluorescence and aggregation-related emissions, respectively. Upon exposure to ethanol, destabilization of the CPN micelle structure and enhanced aggregation were observed, leading to pronounced changes in the emission profile. Notably, the emission intensity ratio (*I*_6__7__9_/*I*_8__4__6_) showed a strong linear correlation with the water concentration, enabling precise calibration for water detection and achieving limit of detection (LOD) and limit of quantification (LOQ) values of 0.4 and 1.3% (v/v), respectively. These findings demonstrate the strong potential of PCPDTBT CPNs as a sensitive and practical platform for the future development of analytical tools to assess biofuel quality and detect water adulteration in ethanol.

## Graphical abstract

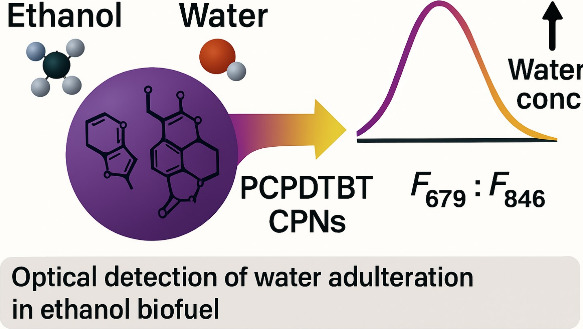

## 1 Introduction

Ethanol is a renewable fuel that offers environmental advantages over fossil fuels, such as lower carbon dioxide emissions, a major contributor to the greenhouse effect.^1–3^ Ethanol is an organic compound containing hydroxyl groups with high miscibility with water, making adulteration both easy to carry out and difficult to detect without laboratory analysis.^4^

According to the Brazilian National Petroleum and Natural Gas Agency (ANP), of the 21,060 samples of ethanol collected in 2021 (pandemic period) from different gas stations, 3% (632) presented unconformities associated with the water content in ethanol, which presents an approximately100% increase in the percentage of nonconforming samples, compared to 2018.^5^

For the detection of adulteration in fuels, many spectroscopic techniques are used:^6–8^ near-infrared (NIR) absorption,^9^ FTIR,^10^ Raman scattering,^11^ and fluorescence with the addition of a fluorescent probe.^4^ Methods based on fluorescence are handy due to their simplicity and minimal sample preparation for analysis, in addition to their high sensitivity in detecting very low concentrations of the analyte. For this reason, many materials are used as fluorescent probes for detection, such as rare-earth-doped materials,^12^ dyes,^13^ conjugated polymer (CP),^14^ and conjugated polymer nanoparticles (CPNs).^15^

CP and CPNs are exciting materials with the electrical characteristics of semiconductors (also called conducting polymers). These materials present π-delocalized electrons in their structure, which leads to interesting optical properties such as high absorption cross-section, intense fluorescence emission, and in many cases, high quantum efficiency, resistance to photobleaching,^16^ and tunable absorption and emission bands depending on the chemical environment in which they are dissolved (as polymers) or suspended (as nanoparticles).^17,18^

CPNs are synthesized by many techniques, such as nanoprecipitation,^19,20^ mini-emulsion,^21^ and coprecipitation,^22^ and they can be suspended in some solvent, forming a colloidal solution. CPN formation requires a change in the physical conformation of the backbone structure, which leads to shifts in its absorption and emission bands compared to those of the free polymer. As a result of these properties, the changes can vary when the same CP is used to form thin films, nanostructures or when the colloidal solution is adulterated by adding some solvent with different polarities.^23^

These properties allow the use of CP and CPNs in photonics, light-emitting diodes,^24^ solar cells,^25^ biology, bacterial agents, and bioimaging.^21,23^ These materials are also widely applied in forensic science as probes in the detection of poisons^26^ and explosives,^27^ enhancement of latent fingerprints,^28^ and detection of DNA.^29^

In this work, we report the changes in the optical properties of the CPN poly­[2,6-(4,4-bis­(2-ethylhexyl)-4*H*-cyclopenta-[2,1-b;3,4-b0]­dithiophene)-*alt*-4,7-(2,1,3-benzothiadiazole)] (PCPDTBT) when ethanol blends with 0 to 16% of water (v·v^–1^) are added to the samples to promote changes in the CP backbone, which are reflected in their fluorescence profiles. Despite significant advances in fluorescence-based sensing for detecting water in ethanol, most reported systems rely on molecular dyes or inorganic nanostructures that require complex synthesis routes, surface functionalization, or exhibit limited stability in polar organic media.^4,6,8^ Additionally, many optical probes lack dual-emission features that enable self-referenced measurements, leading to reduced accuracy under fluctuating experimental or environmental conditions.^30^ To date, no studies have explored the use of conjugated polymer nanoparticles (CPNs), particularly PCPDTBT-based systems, for ratiometric optical detection of water content in ethanol. This work addresses this gap by demonstrating, for the first time, the proof-of-concept application of PCPDTBT CPNs as fluorescent nanosensors for monitoring water adulteration in ethanol, establishing the foundation for future analytical tools in biofuel quality control.

## 2 Materials and Methods

### 2.1 Materials

Poly­[2,6-(4,4-bis­(2-ethylhexyl)-4*H*-cyclopenta-[2,1-b;3,4-b0]­dithiophene)-*alt*-4,7-(2,1,3-benzothiadiazole)] (PCPDTBT), and Tween 20 were purchased from Sigma-Aldrich and used as received. Tetrahydrofuran (THF) was purchased from IMPEX (PA grade). Deionized water was collected from a reverse osmosis water purifier filter, model Evolution RO0410UF.

### 2.2 Preparation and Characterization of CPNs

PCPDTBT CPNs were prepared using the nanoprecipitation method.^20,31^ 10.0 mg of PCPDTBT CP was dissolved in 4.0 mL of THF, yielding a stock solution at 2.5 mg·mL^–1^. Nanoparticle production was performed by initially preparing two solutions: the organic solution and the aqueous solution. The organic phase consists of 180 μL of the CP stock solution dissolved in 820 μL of THF, and the aqueous phase consists of 8.8 mL of deionized water and 1.2 mL of Tween 20 (a stock solution of 10 mg·mL^–1^ H_2_O). Then, the aqueous phase was stirred, and the organic phase was added dropwise. The mixed solution was stirred for 24 h to evaporate THF. After that, the final solution was filtered through filter paper C42. The water lost during the stirring and filtration procedures was replaced to obtain a final volume of 10 mL, and an external calibration procedure determined the final concentration. After preparation, the samples were stored at 4 °C in a refrigerator. Under these conditions, the colloidal solution remains stable for up to 8 weeks.^31^

The hydrodynamic diameter of CPNs was obtained at room temperature by dynamic light scattering (DLS) analysis using a Zetasizer NanoZS (Malvern Instruments Ltd., UK).

Field Emission Gun Scanning Electron Microscopy (FEG-SEM) images of CPNs were collected. Twenty mL of PCPDTBT CPNs and CPNs mixed with ethanol was deposited onto silicon substrates and dried at room temperature for 15 min. The substrates were then sputter-coated with a 10 nm gold layer. The nanoparticles and aggregates were examined using a FEG-SEM Mira 3 (Tescan) operating at 5 kV. For EDS measurements, an XFlash 6160 detector (Bruker) was used at 10 kV, and EDS mapping was performed with the Quantax Esprit software.

Atomic Force Microscopy (AFM) images were also obtained. A volume of 10 μL of PCPDTBT NPs diluted 1:10 in water and 10 μL of PCPDTBT NPs diluted in 60% (v/v) ethanol (collected directly from a gas station – GSEthanol) were deposited onto silicon substrates. For sample drying, the deposited PCPDTBT NP solutions were kept on the substrates for 24 h at room temperature (25 °C) inside a sealed Petri dish protected from light. Topographical images with atomic resolution were obtained using an AFM Workshop TT2 microscope in noncontact (vibrating) mode to study morphology. The scans were performed using an aluminum-coated silicon cantilever with a spring constant of 5.0 N/m and a resonance frequency of 160 kHz.

X-ray diffraction (XRD) measurements were performed using an Aeris Cement (Marvern Panalytical) diffractometer operating in a scanning line detector (1D) mode. Approximately 200 μL of each sample — PCPDTBT CPNs dispersed in water and CPNs dispersed in ethanol were drop-cast onto a zero-background silicon substrate and allowed to dry under ambient conditions before analysis. The diffraction patterns were collected over 2θ from 5° to 80°, with a step size of 0.0217° and a counting time of 400 s per step (scan speed of 0.0138°/s). The incident beam was conditioned with a nickel β-filter, Soller slits of 0.04 rad, a 1/2° divergence slit, and a fixed beam mask of 13 mm. The diffracted beam path employed an antiscatter slit of 9 mm, with no additional β-filter or Soller slits. The sample stage was rotated at 0.5 rps during the scan to improve particle orientation statistics.

Fourier-transform infrared (FTIR) absorption spectra were recorded using a PerkinElmer Spectrum 100 spectrophotometer equipped with an ATR accessory. The samples were drop-cast directly onto the ATR crystal and dried at room temperature before measurement. For each spectrum, 8 successive layers of 20 μL were deposited for PCPDTBT, CPNs, and CPN + Ethanol samples. The Tween 20 spectrum was also acquired in its liquid form.

### 2.3 Optical Measurements

Five mL of PCPDTBT CPNs were centrifuged at 5000 rpm for 15 min prior to the optical analysis. The supernatant was then collected for absorption and fluorescence measurements. The UV–vis absorption measurements were performed by an Ocean Optics spectrometer coupled to a deuterium-tungsten lamp by optical fibers (Ocean Optics TP 300 UV–vis). The samples were inserted into a 1 cm path length cuvette. The light from the lamp was directed to the sample passing through the cuvette and collected by the second optical fiber, which was coupled to the Ocean Optics HR4000 spectrometer. All the measurements were taken with an integrating time of 3.8 ms, 50 acquisitions per spectrum (average), and a boxcar smooth of 5 points. The absorbance measurements in ethanol were performed by adding 1 mL of PCPDTBT CPNs dispersion into a quartz cuvette (1 cm optical path length), followed by the successive addition of 500, 1000, 2000, and 3000 μL of ethanol (Synth absolute ethanol, 99.5%). After each ethanol addition, the mixture was allowed to equilibrate for 2 min before the absorbance spectrum was recorded.

For the emission measurements, simulated adulterated ethanol blends were prepared with Synth absolute ethanol (99.5%) and deionized water from a reverse osmosis water purifier (Evolution RO0410UF). The blends were prepared with 0, 4, 8, 12, and 16% of water in ethanol (v·v^–1^) and labeled according to the water content: Ethanol 0, Ethanol 4, Ethanol 8, Ethanol 12, and Ethanol 16. Then, 1.0 mL of PCPDTBT CPNs were inserted in a quartz cuvette (1 cm internal path length), and 2 mL of each blend was directly inserted in the cuvette in steps of 200 μL. For each step, an emission spectrum was recorded. The emission spectra at different ethanol contents were collected by exciting samples (CPN and blends) in a quartz cuvette with 405 nm diode laser (37 mW). To avoid photodegradation, the laser beam was focused 1 cm outside the cuvette, and after each measurement, the beam was interrupted to add 200 μL of ethanol blends. An Ocean Optics spectrometer (HR4000) coupled with an optical fiber (Ocean Optics TP 300 UV–vis) collected the fluorescence spectra as a function of the ethanol blends in a front-side configuration. Each spectrum resulted from 100 ms of integrating time and 50 acquisitions per average spectrum. The emission ratio was calculated by directly integrating the areas under each curve. For the curve at 679 nm, integration was performed from 600 to 750 nm, and for the emission band at 846 nm, from 750 to 1000 nm. After integration, the area values were normalized by the total volume added in the cuvette, and the emission ratio *I*_679_/*I*_846_ was calculated.

Fluorescence lifetime (FLT) measurements were also performed using a FluoTime 100 spectrofluorometer (PicoQuant, Berlin, Germany) operating in the time-correlated single-photon counting (TCSPC) mode. PCPDTBT nanoparticles were excited with a pulsed laser at 630 nm, and the time evolution of the emission intensity was recorded within a 100 ns time window. To suppress the excitation light before the emission monochromator, an optical filter (Filter #4, PLS 600) was inserted in the detection path. All measurements were carried out in a 1 cm path length quartz cuvette with four polished faces.

## 3 Results and Discussion

Figure 1 presents the absorption (black dotted curve) and emission spectra (blue line) of PCPDTBT CPNs. PCPDTBT CPNs present a broad absorption band from 250 to 900 nm. The absorption peak is about 680 nm, approximately 20 nm blue-shifted from the PCPDTBT CP in THF (inset of Figure 1). This shift indicates the formation of the CPNs after synthesis, and it is attributed to the alteration of the polymer chain conformation during the nanoparticle formation.^21^ PCPDTBT CP also tends to form aggregates. The shoulder around 750–800 nm (arrow) indicates the presence of aggregates in the CPNs sample, remaining in the filtration process.^32^ When the sample was excited at 405 nm, the emission spectrum of the PCPDTBT CPNs exhibits two prominent maxima at 679 and 846 nm, indicating the presence of CPNs and aggregates after the synthesis.

**Figure 1:**
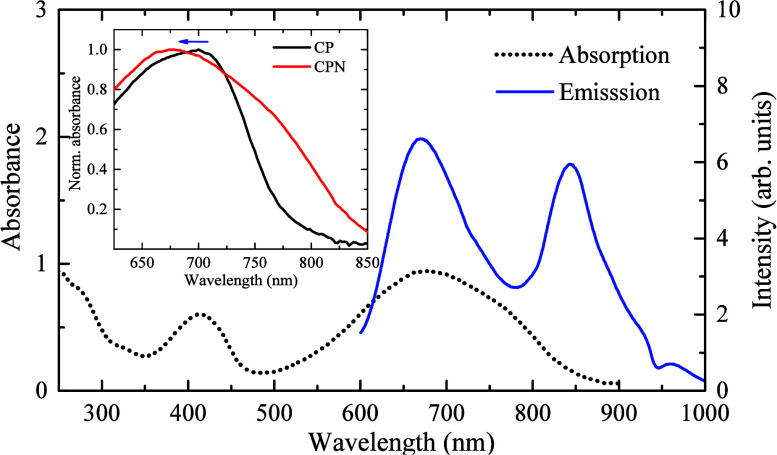
Absorption and emission of PCPDTBT CPNs (19.9 μg·mL^–1^). Emission was recorded at 405 nm excitation.

For the most precise identification of the aggregate absorption and emission bands on the studied CPNs sample, 5 mL of the PCPDTBT CPNs were centrifuged at 5000 rpm, and the absorption and emission bands were measured in both cases before (Figure 1) and after the centrifugation and the separation of the supernatant (majority nanoparticles). Figure 2 presents the normalized absorption, emission, and subtracted spectra from centrifugation. Subtracting both spectra reveals the location of the aggregates band in PCPDTBT CPNs, with absorption around 700–800 nm and emission mainly in the near-infrared range (800–1000 nm). Despite overlap between the emission bands, this result indicates that the emission band centered at 679 nm is mainly due to PCPDTBT CPNs. In comparison, the band around 846 nm is attributed to aggregates that can pass through the filtration step during synthesis.

**Figure 2:**
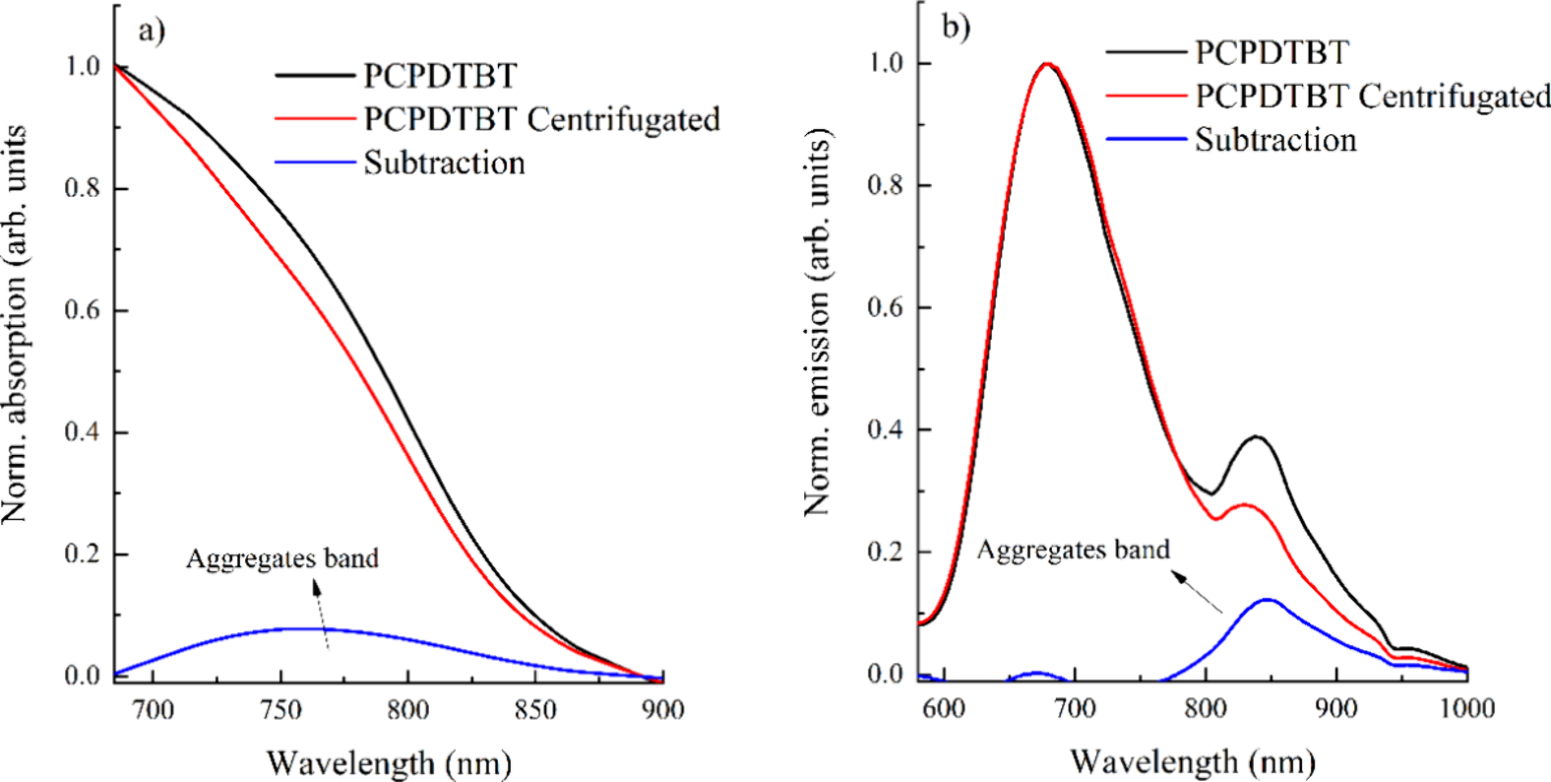
Normalized (a) absorption and (b) emission of the PCPDTBT CPNs as prepared (black lines), after centrifugation (red lines), and subtraction (blue lines), showing the position of aggregate bands.

The studied CPNs were formed in aqueous media containing CP, water, and Tween 20 to stabilize the micelles. Ethanol (CH_3_CH_2_OH) is an organic compound, and its addition to the sample will alter the chemical environment around the CPN, which may destabilize the micelle and promote aggregate formation. This aggregate formation will change the ratio of the emission bands at *I*_679_/*I*_846,_ which can be used as an indicator of ethanol presence.

Figure 3 presents the effect of adding absolute ethanol to the PCPDTBT CPNs. As observed in Figure 3a, the absorption band increases in the aggregate’s region, and the emission band of PCPDTBT CPNs (Figure 3b) completely vanished after additions higher than 600 μL (60% of the initial volume of CPNs) of absolute ethanol. These results indicated the possible destabilization of the CPNs micelles. Once ethanol contains a certain amount of water, it is likely that by increasing the water content in ethanol, the destabilization of the micelle decreases, which is also reflected in the emission ratio *I*_679_/*I*_846_ (inset of Figure 3b). This effect can be used to construct a calibration curve to estimate the water content in ethanol blends.

**Figure 3:**
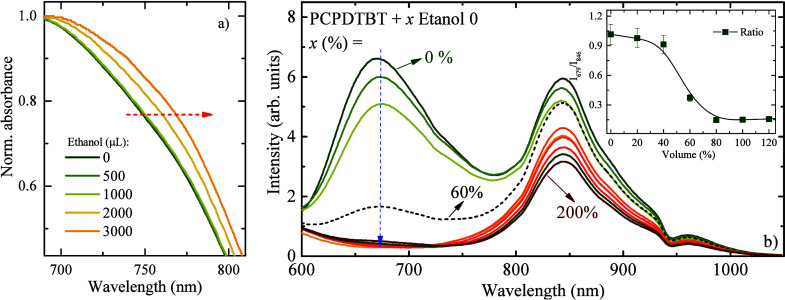
(a) Normalized absorption of PCPDTBT CPNs showing increased absorbance in the aggregate region with increasing ethanol content and (b) emission spectra excited at 405 nm with the addition of absolute ethanol. The inset shows the ratio of the integrated areas of the emission band at 679 and 846 nm (*I*_679_/*I*_846_).

To prove the concept and investigate the possible changes in the emission spectra and the emission ratio *I*_679_/*I*_846_, five ethanol blends were performed with 0, 4, 8, 12, and 16% of water (labeled Ethanol 0–16) and inserted in 1 mL of the sample of PCPDTBT CPNs (19.9 μg·mL^–1^), from steps of 200 μL for each step, an emission spectrum was collected from 600 to 1000 nm (Figure 4).

**Figure 4:**
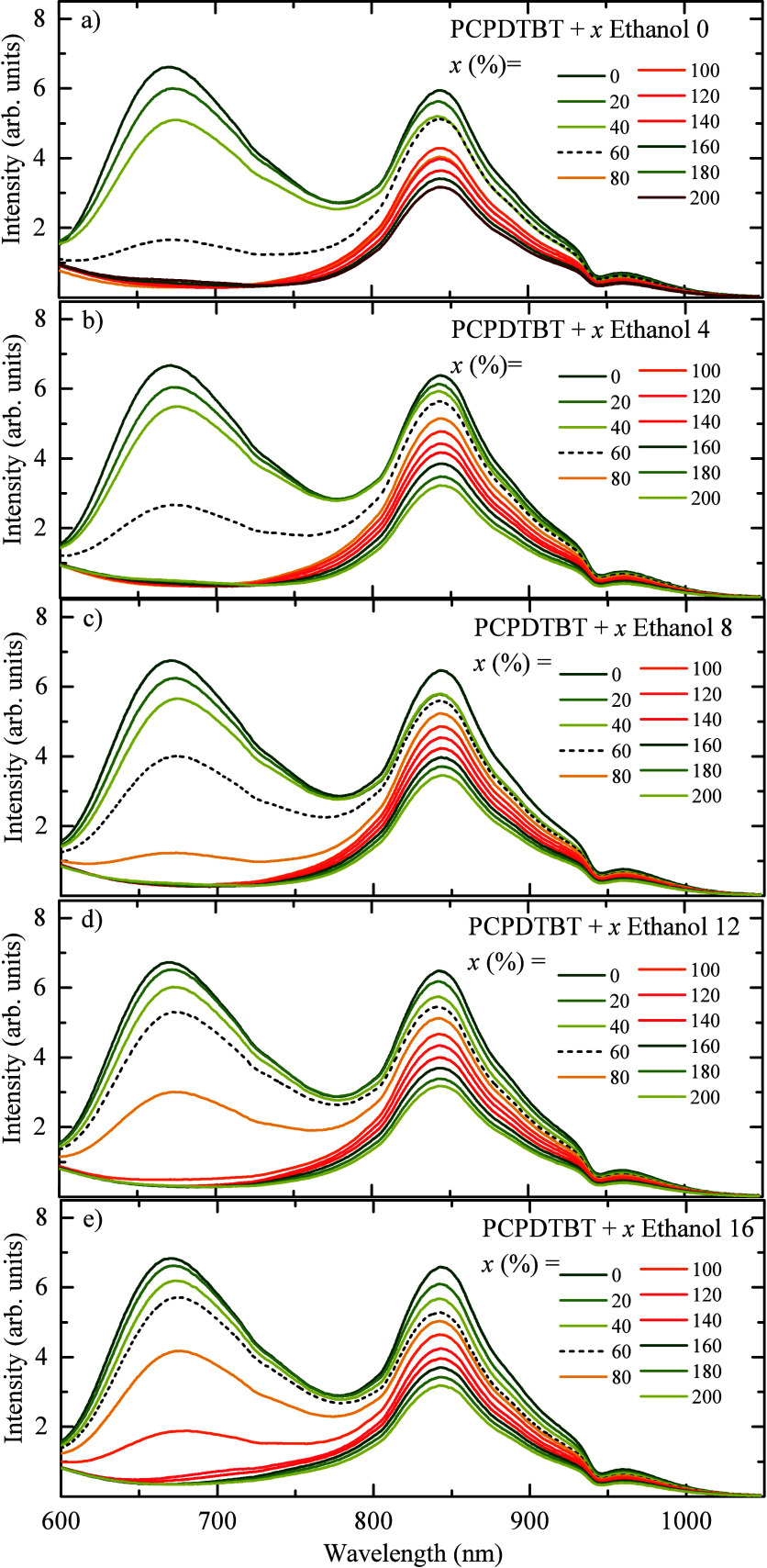
Emission spectra of the PCPDTBT CPNs excited at 405 nm for different ethanol blends: (a) Ethanol 0, (b) Ethanol 4, (c) Ethanol 8, (d) Ethanol 12, and (e) Ethanol 16. The dashed lines represent the spectrum obtained after adding 600 μL (60%) to the ethanol blends.

Figure 4 shows the emission of the samples excited at 405 nm, as reported earlier in Figure 3. The CPNs emission band at 679 nm decreases more rapidly than the band at 830 nm with the addition of ethanol. However, as the water content in the ethanol blends increases, the emission band shows different behavior. Comparing the emission curves to a fixed volume of 60% (600 μL of Ethanol 0–16) (Figure 4a–e), we observe an increase in the emission band as the water present in the blends increases.

Figure 5a presents the emission ratio *I*_679_/*I*_846_ from the data shown in Figure 4a–c. The ratio curves shift with increasing water content, indicating that the destabilization of the micelle is reduced by adding water to blends, leading to lower aggregation. Figure 5b shows the ratio for a fixed added volume in the samples (1 mL of sample and 600 μL of ethanol blends, corresponding to 60% of the initial volume) as a function of water content in the ethanol blend. As observed, the ratio increases linearly with water content, allowing it to be used to specify the water content in the ethanol blends. According to IUPAC recommendations, the LOD and LOQ were determined using the slope of Figure 5b and the standard deviation of the blank (ethanol with 0% water).^33^ The obtained LOD and LOQ values were 0.4% and 1.3%, respectively.

**Figure 5:**
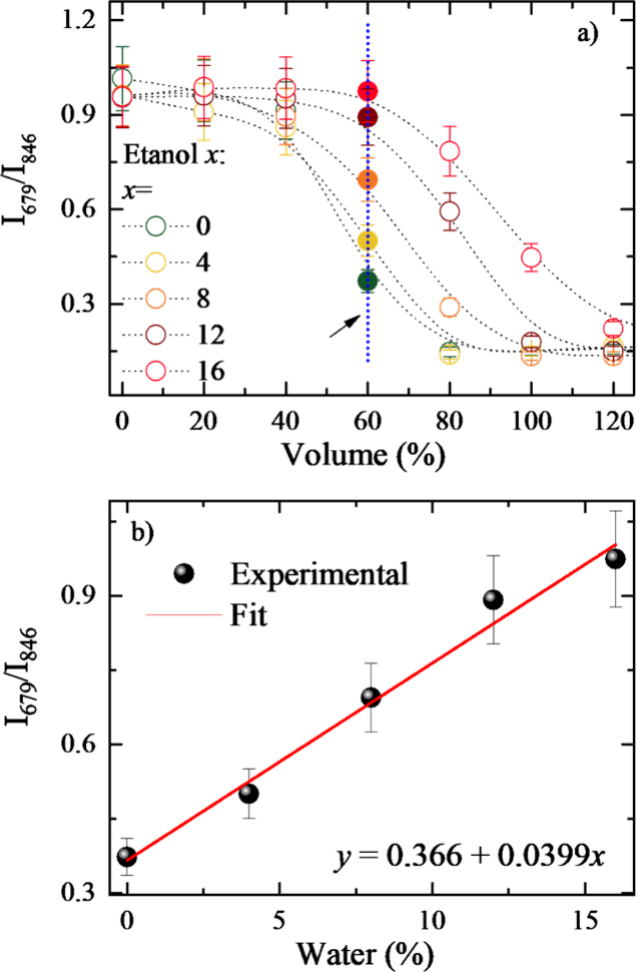
(a) Emission ratio (*I*_679_/*I*_846_) derived from Figure 4 for different ethanol blends and (b) emission ratio for a fixed ethanol blend volume (600 μL, corresponding to 60% of the initial volume of PCPDTBT CPNs) as a function of the water content (*R* ^2^ = 0.98513).

To evaluate the use of the PCPDTBT CPNs and the emission ratio *I*_679_/*I*_846_ as a function of water content in ethanol blends, an adding/recovery test was performed in three ethanol blends prepared with 12 ± 1%. The spectra and the corresponding percentages are shown in Figure 6. As observed, there is a slight shift in the emission band, mainly at 679 nm, with the addition of the ethanol blends. The average percentage found was 13 ± 1%, which corresponds to a recovery of 108%, indicating a good percentage of recovery, between 90 and 110%.^4^ This result indicates that the PCPDTBT CPNs aggregation effect upon ethanol addition can be used to estimate the water content in ethanol blends.

**Figure 6:**
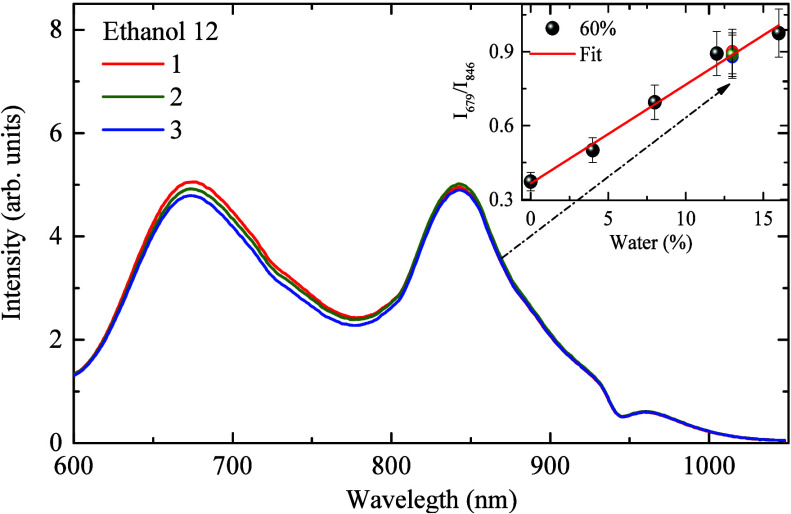
PCPDTBT emission spectra after adding 600 μL of Ethanol 12 measured in triplicate. The inset presents the recovery percentage as a function of the emission ratio.

Compared to other nanoparticle probes reported for ethanol–water or biofuel analysis, the PCPDTBT CPNs exhibit a distinctive dual-emission ratiometric response (*I*_6__7__9_/*I*_8__4__6_) arising from nanoparticle fluorescence and aggregation-related near-infrared emission. This property enables internal calibration and minimizes environmental and instrumental fluctuations. Recent dual-emission ratiometric systems report limits of detection for water in organic solvents (including ethanol) in the 0.003–0.3% (v/v) range, demonstrating that dual-emission probes can reach LODs well below 1% depending on the probe design and readout channel.^30^ For instance, rhodamine-based fluorescent nanoparticles used by Passos et al. (2021) achieved a limit of detection (LOD) of 0.8% (v/v) water in ethanol,^4^ whereas the PCPDTBT CPNs developed here present an improved LOD of 0.4% (v/v) under similar conditions. Furthermore, unlike metal–organic framework (MOF) nanoparticles, such as the mixed-lanthanide system reported by Li et al. (2019),^6^ which require multistep synthesis and coordination control, PCPDTBT nanoparticles are obtained through a simple nanoprecipitation method and remain stable for up to 8 weeks. Overall, the PCPDTBT CPNs combine the synthetic simplicity of organic probes with the sensitivity and reproducibility of nanostructured optical sensors, making them a promising alternative to previously reported dye-doped, or MOF-based, nanoparticle systems for ethanol–water detection.

In addition, to confirm the hypothesis that the emission ratio (*I*_679_/*I*_846_) change is a result of the destabilization of the CPNs of PCPDTBT promoted by aggregate formation with addition of Ethanol, dynamic light scattering (DLS) and Field Emission Gun Scanning Electron Microscope (FEG-SEM) measurements were taken for PCPDTBT CPNs as synthesized and after the addition of ethanol until the complete vanishment of the 679 nm emission.

In Figure 7a, the PCPDTBT CPNs exhibit a Gaussian distribution with an average size of 154 ± 44 nm, and a second, broader distribution band with low intensity is observed above 300 nm, attributed to aggregates in the sample. In Figure 7b, adding ethanol until the disappearance of the emission band at 679 nm, a high reduction of the first Gaussian located at 154 nm and an increase in particle size distribution above 500 nm are observed, indicating the aggregation formation, which confirms the indication of this phenomenon by the previous results from Figures 3–5. The PDI index also shows an increase from 0.5 ± 1 to 0.8 ± 0.1, and Zeta potential from −10 ± 2 mV to −12.8 ± 0.5 mV.

**Figure 7:**
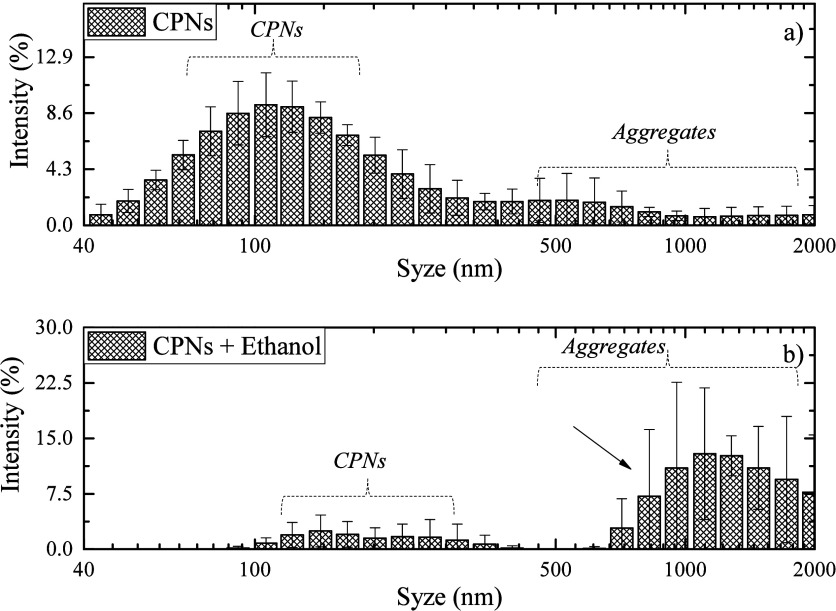
DLS measurement of PCPDTBT CPNs: (a) as prepared and (b) after adding 1.0 mL of absolute ethanol (Ethanol 0).

Figure 8b,d shows the morphology of the nanoparticles and their aggregates, respectively. As observed, the nanoparticles exhibit a morphology that is reported by Caires (2024) for PCPDTBT CPNs.^31^ Upon the addition of ethanol, clear morphological changes were observed. The nanoparticle size distribution obtained from SEM data revealed a mean size of 172 ± 5 nm, whereas after ethanol addition, the resulting major structures ranged from 0.3 to 4.0 μm.

**Figure 8:**
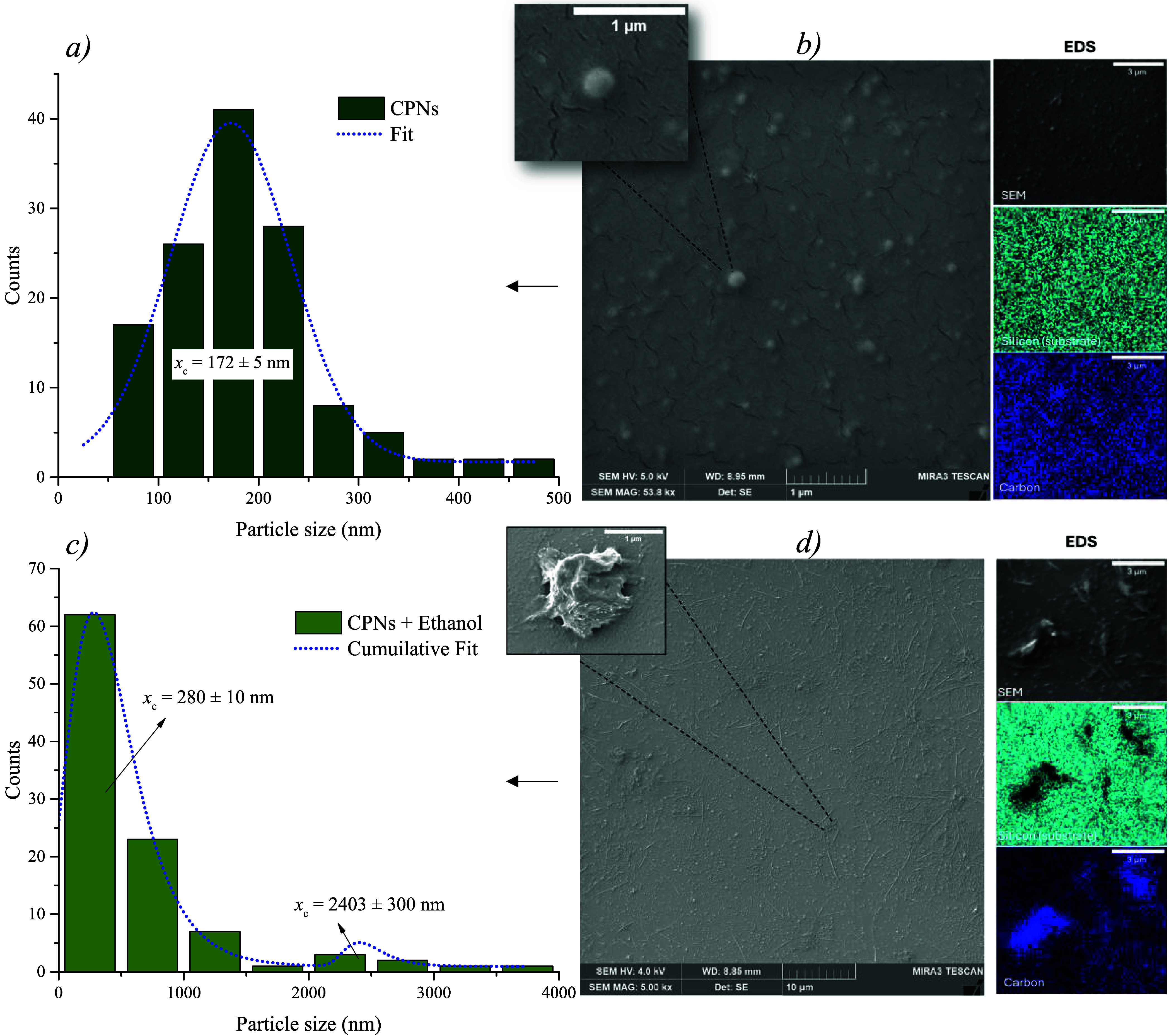
(a) Particle size distribution diagram and (b) FEG-SEM images of PCPDTBT CPNs dispersed in water and (c) particle size distribution diagram and (d) FEG-SEM images of PCPDTBT CPNs dispersed in ethanol. Insets in both images show representative nano- and microstructures observed across the substrate; each inset includes a 1 μm scale bar.

Additionally, X-ray Diffraction (XRD) and fluorescence lifetime (FLT) measurements were also collected for PCPDTBT CPNs dispersed in water and in ethanol (Figures S2 and S3 in the Supporting Information, respectively). The XRD data revealed a diffraction peak at 2θ ≈ 27.5° that can be attributed to the π–π stacking of conjugated polymer backbones.^34,35^ In the presence of poor solvents such as ethanol, planar aggregation is promoted. The slight shift observed in the ethanol-containing sample suggests tighter molecular packing induced by solvent evaporation. Although Tween 20 can exhibit weak reflections in the 19–28° range when dried, its contribution here is likely negligible due to the absence of additional crystalline peaks typically associated with this surfactant. From the FLT results, a lifetime reduction from 2.48 to 1.30 ns was observed upon the addition of ethanol, reinforcing the formation of aggregates when PCPDTBT CPNs are subjected to ethanol. This could be attributed to the reduced intra- and intermolecular interactions of CPNs when compared to the aggregated state, which usually leads to a decrease in fluorescence lifetime.^36,37^

A preliminary test using samples in triplicate collected directly from a gas station was performed to evaluate changes in hydrodynamic size in 60% (v/v) ethanol (labeled GSEthanol) through DLS measurements (Figure 9). For the PCPDTBT CPNs in water, the average particle size distribution is located at 184 ± 5 nm, whereas the samples dispersed in gas station ethanol (GSEthanol) exhibit a shift toward larger hydrodynamic sizes, centered at 593 ± 10 nm. This shift indicates an aggregation process, also observed with absolute ethanol (Figure 7). In addition, Figure S4 in the Supporting Information presents AFM characterization of PCPDTBT CPNs and CPNs + GSEthanol, confirming that larger structures are formed following the addition of bioethanol.

**Figure 9:**
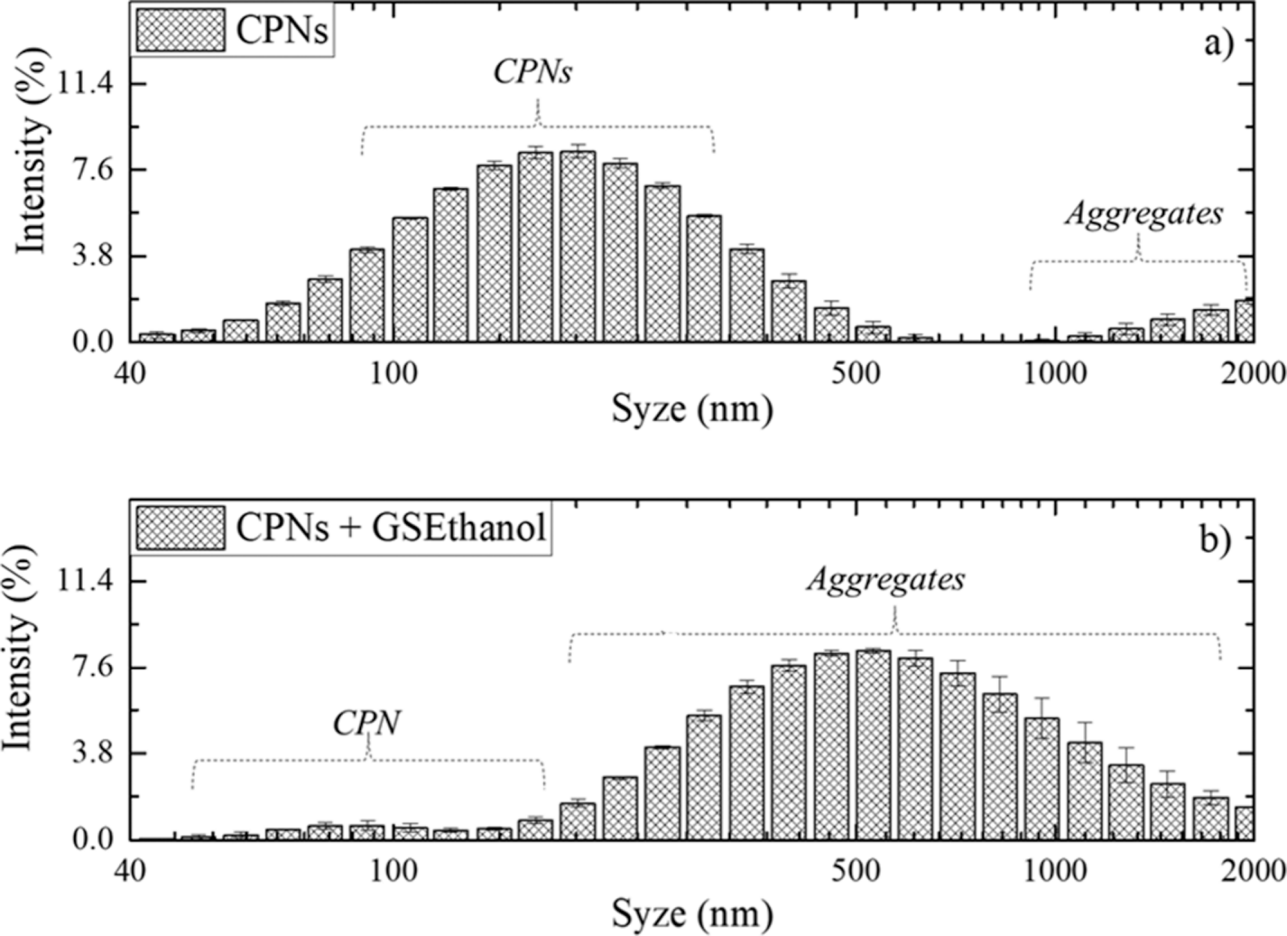
(a) Hydrodynamic size of PCPDTBT CPNs and (b) CPNs dispersed in 60% (v/v) ethanol collected directly from a gas station (GSEthanol).

The findings suggest that the distinctive optical response of PCPDTBT CPNs to ethanol–water mixtures can be further exploited to develop a robust analytical methodology for quantifying water adulteration in ethanol biofuels.

## 4 Conclusions

In conclusion, the synthesized PCPDTBT conjugated polymer nanoparticles demonstrated significant changes in their optical absorption and emission profiles in response to water adulteration in ethanol blends. The addition of water to ethanol blends reduced this aggregation, allowing a linear relationship between the emission ratio (*I*_679_/*I*_846_) and water content to be established. This behavior indicates the potential of PCPDTBT CPNs as a fluorescent nanosensor for detecting water adulteration in ethanol biofuels. This approach offers a rapid, sensitive, and practical tool for monitoring fuel adulteration, thereby improving quality control and compliance with regulatory standards in biofuel production and distribution. As part of our future work, we will systematically evaluate PCPDTBT CPNs behavior in commercial ethanol biofuel samples with characterized impurity profiles to quantify the possible synergistic or antagonistic effects of these byproducts on the sensing performance.

## Supplementary Material



## Data Availability

The data are not publicly available due to intellectual property protection but are available from the corresponding author upon reasonable request

## Notes

FTIR spectra and vibrational assignments of PCPDTBT conjugated polymer nanoparticles (CPNs) and CPNs dispersed in ethanol; XRD patterns of CPNs in water and ethanol; fluorescence decay curves; and AFM images showing morphological changes upon ethanol addition (PDF)
