# Supplementary material for: Optical Detection of Water Adulteration in Ethanol Using PCPDTBT-Conjugated Polymer Nanoparticles
Source: ACS Omega. 2026 Jan 10;11(3):3965–73. doi: 10.1021/acsomega.5c07266 (PMC12854640; doi:10.1021/acsomega.5c07266)
Supplement: Supplementary file 1 [file ao5c07266_si_001.pdf]

Optical Detection of Water Adulteration in Ethanol Using PCPDTBT Conjugated Polymer Nanoparticles

Claudio Y. Morassuti[1,2], Leandro O. Araújo[1], Samuel L. Oliveira[1], Anderson R. L. Caires[1,*]

[1] Grupo de Óptica e Fotônica, Instituto de Física, Universidade Federal de Mato Grosso do Sul, 79.070-900, Campo Grande, MS, Brazil.

[2] Grupo de Biofabricação, Centro de Tecnologia da Informação Renato Archer, 13.069-901, Campinas, SP, Brazil.

[*] *Corresponding author: anderson.caires@ufms.br*

Supporting Information

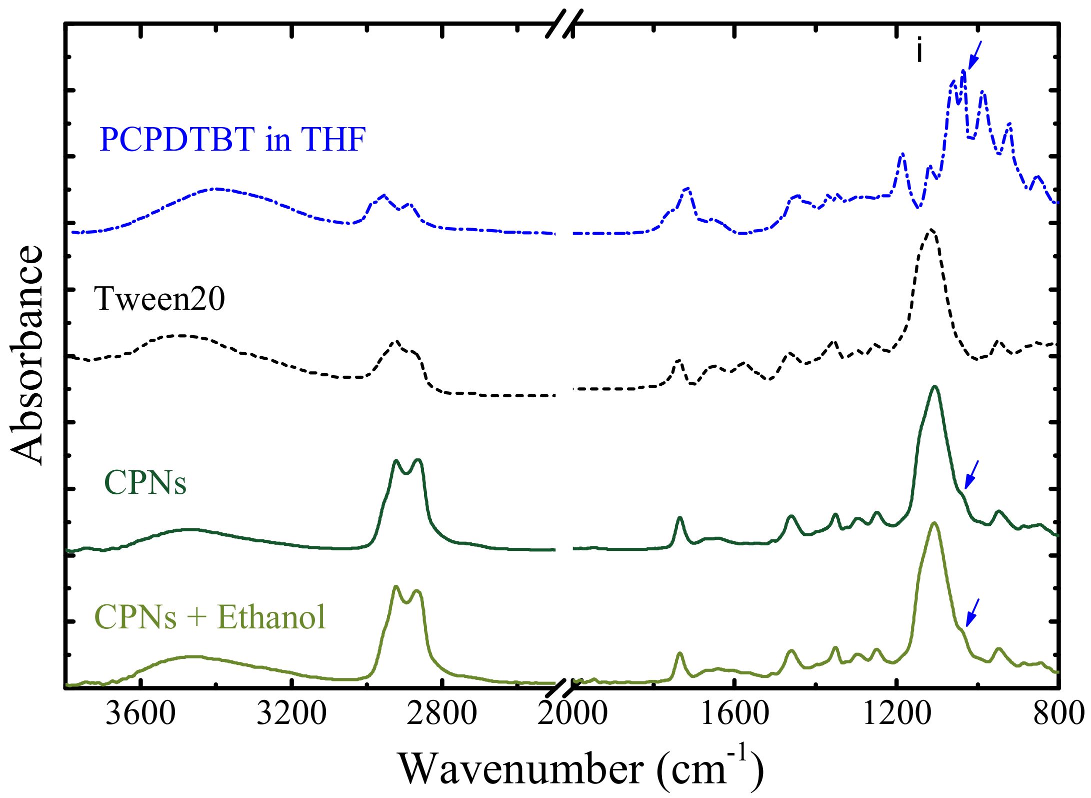

Figure S1. FTIR characterization was performed for the PCPDTBT CPNs, CPNs dispersed in ethanol, Tween 20, and PCPDTBT polymer. All spectra were recorded after drying at room temperature. Table S1 lists the main absorption bands and their corresponding vibrational assignments. Arrows mark the PCPDTBT-related vibrational band detected in both systems (CPNs and CPNs + Ethanol).

Table S1. FTIR absorption peaks found in CPNs and CPNs + Ethanol samples.

**Position (cm^{-1})**	**Vibrational mode**	**Assignment**	Ref.
**3470**	O–H stretching	Stretching vibration of hydroxyl groups (residual water or Tween 20 hydroxyl groups).	41
**2924**	C–H asymmetric stretching	Asymmetric stretching of methyl and methylene groups (–CH_2–, –CH_3) from Tween 20 chains.	42

**2863**	C–H symmetric stretching	Symmetric stretching of methyl and methylene groups.	43
**1740**	C=O stretching	Carbonyl (ester) stretching vibration of Tween 20.	41,42
**1626**	C=C stretching (aromatic)	Vibration of the conjugated backbone of PCPDTBT.	43,44
**1461**	CH_2 scissoring	Bending vibration of methylene groups in the surfactant chain.	41,44
**1354**	C–H bending	Deformation vibration of aliphatic C–H bonds.	41,43
**1297**	C–O stretching	Ester C–O stretching vibration.	43
**1251**	C–O–C asymmetric stretching	Asymmetric stretching of ether groups (Tween 20).	36,42
**1104**	C–O–C symmetric stretching	Symmetric stretching of ether groups (Tween 20).	36,42

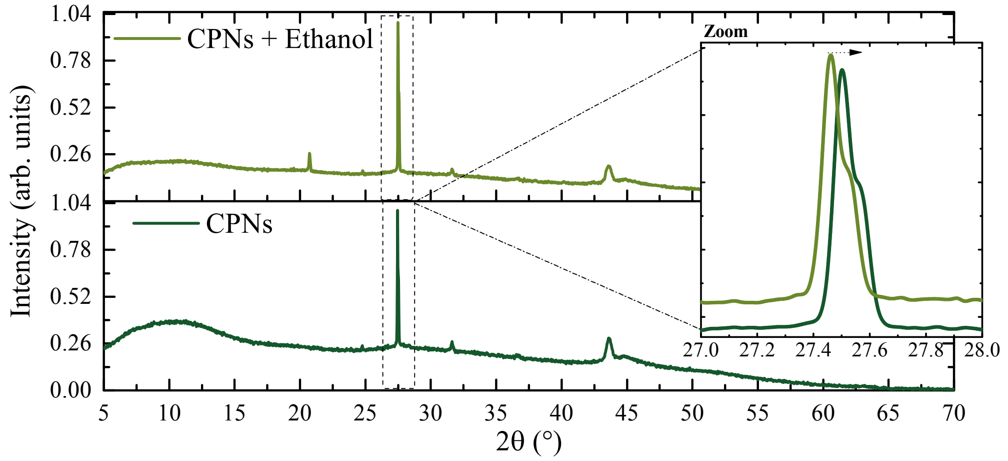

Figure S2. XRD patterns of PCPDTBT nanoparticles dispersed in water (CNPs) and in absolute ethanol (CPNs + Ethanol). The inset highlights the shift of the diffraction peak near 27.5°.

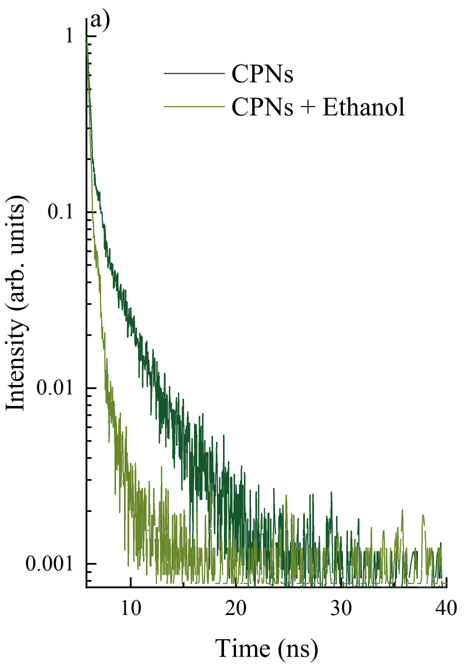

Figure S3. Fluorescence decay curves of CPNs and CPNs dispersed in ethanol (200% v.v^{-1}).

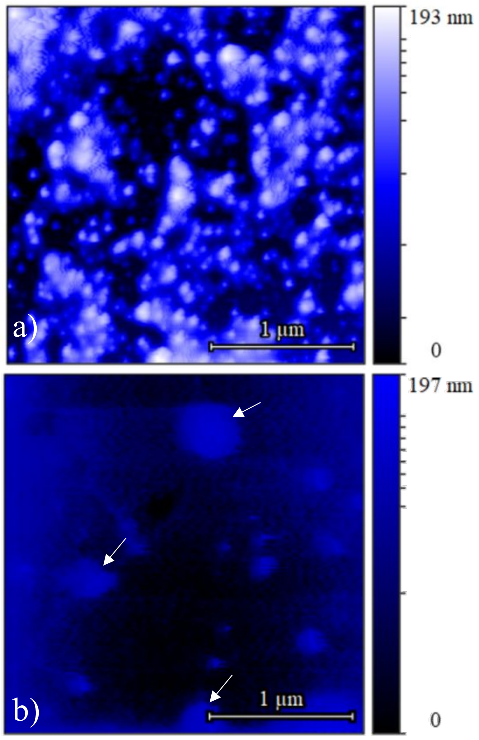

Figure S4. AFM images of (a) CPNs dispersed in water and (b) CPNs dispersed in 60% (v.v^{-1}) ethanol. Arrows indicate larger structures observed upon the addition of GSEthanol.
